# Effect of adipose-derived mesenchymal stem cell transplantation on vascular calcification in rats with adenine-induced kidney disease

**DOI:** 10.1038/s41598-017-14492-9

**Published:** 2017-10-25

**Authors:** Shinya Yokote, Yuichi Katsuoka, Akifumi Yamada, Ichiro Ohkido, Takashi Yokoo

**Affiliations:** 1 Division of Nephrology and Hypertension, Department of Internal Medicine, The Jikei University School of Medicine, 3-25-8 Nishi-shimbashi, Minato-ku, Tokyo, 105-8461 Japan; 2 Department of Urology, St. Marianna University School of Medicine, 2-16-1, Sugao, Miyamae-ku, Kawasaki, Kanagawa 216-8511 Japan; 3 Department of Pediatrics, The Jikei University School of Medicine, Tokyo, Japan

## Abstract

Previous studies have investigated the use of mesenchymal stem cells (MSCs) to treat damaged kidneys. However, the effect of adipose-derived MSCs (ASCs) on vascular calcification in chronic kidney disease (CKD) is still poorly understood. In the present study, we explored the potential of ASCs for the treatment of CKD and vascular calcification. CKD was induced in male Sprague-Dawley rats by feeding them a diet containing 0.75% adenine for 4 weeks. ASCs transplantation significantly reduced serum inorganic phosphorus (Pi) as compared to that in the control. The histopathology of the kidneys showed a greater dilation of tubular lumens and interstitial fibrosis in the control group. Calcium and Pi contents of the aorta in the ASCs transplantation group were lower than those in the control group. Von Kossa staining of the thoracic aorta media revealed that ASCs transplantation suppressed vascular calcification. Thus, this study revealed that autogenic ASCs transplantation inhibits kidney damage and suppresses the progression of vascular calcification in the CKD rat model, suggesting that autogenic ASCs transplantation is a novel approach for preventing the progression of CKD and vascular calcification.

## Introduction

Chronic kidney disease (CKD) is a major public health problem worldwide because end-stage renal disease (ESRD) is associated with cardiovascular morbidity and mortality^1,2^. Regardless of advances in supportive therapy for CKD, 2.6 million people received renal replacement therapy worldwide in 2010. Because of the increase in the number of patients with ESRD, the worldwide use of replacement therapy is projected to more than double by 2030^3^. Therefore, new modalities to prevent the progression of CKD and vascular calcification are desperately needed.

In the last few decades, advances in stem cell biology have led to the development of stem cell-based therapies, and many researchers have investigated the potential of stem cells for kidney regeneration^4–11^. We have succeeded in regenerating a functional kidney *de novo* using the kidney development program in rat embryos by applying human mesenchymal stromal cells (MSCs) at the niche of organogenesis^4,5^. Human MSCs are multipotent cells that can be isolated from several tissues and expanded *ex vivo* for clinical use^12,13^. They have also been shown to accelerate recovery and prevent acute or chronic renal failure in multiple disease models^14–19^, thus providing novel strategies for kidney regeneration. MSCs have been reported to enhance angiogenesis^20,21^, and conversely induce vascular remodelling^22^ and calcification in a hyperlipidemic rat model^23^. Furthermore, MSCs alter osteoblast differentiation during metabolic acidosis and uremic conditions^24,25^. Thus, it is important to investigate the effect of MSCs transplantation on vascular calcification in patients with CKD. However, such effects have not been evaluated to date.

The adenine-induced kidney disease model is a well-established and widely used CKD model and is associated with vascular calcification^25–28^, in which chronic tubulointerstitial nephritis is caused by the accumulation of 2,8-dihydroxyadenine crystals in the renal tubules and interstitium. Moreover, because arterial medial calcification develops in adenine-induced CKD rats with hyperphosphatemia, the adenine-induced model has been used as a model of uremic vascular calcification.

In the present study, we investigated the effects of transplanting autologous ASCs into rats with adenine-induced CKD on vascular calcification.

## Results

### MSCs isolation

MSCs were isolated from 9-week-old Sprague-Dawley (SD) rats. Characterization of MSCs cultured in standard culture medium showed a spindle-shaped morphology. Surface markers of MSCs were characterized by flow-cytometric analysis. MSCs were positive for CD90 (97.05 ± 1.25%) and CD29 (97.46 ± 0.25%), and negative for CD31 (0.72 ± 0.26%) and CD45 (1.16 ± 0.20%) (Supplementary Fig. S1A). MSCs had the capacity to differentiate into adipogenic, chondrogenic, and osteogenic lineages (Supplementary Fig. S1B–E).

### MSCs transplantation reduces adenine-induced kidney injury

**Figure 1:**
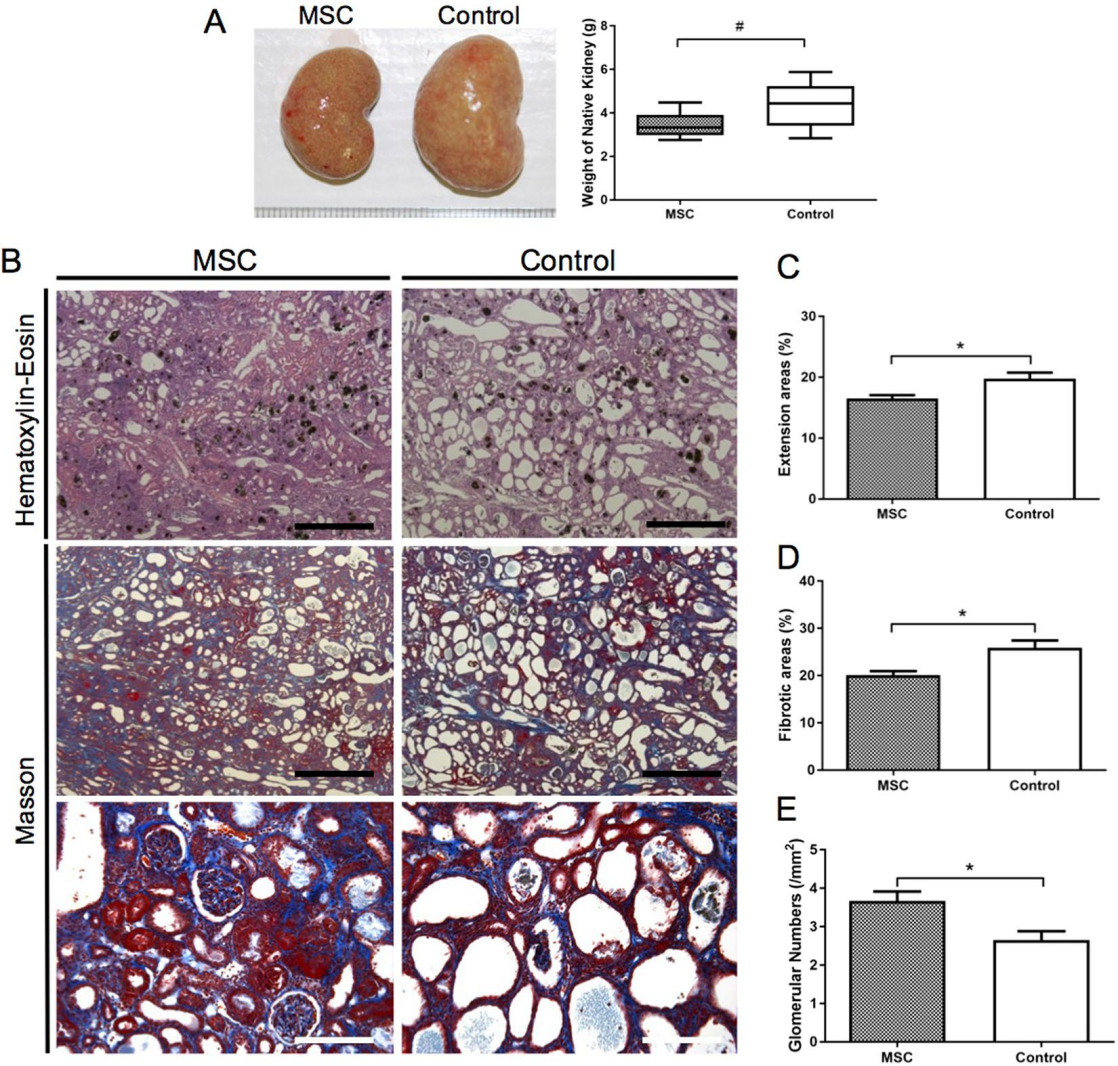
Histopathology of the kidneys in adenine-fed rats. (**A**) The average kidney weight in the control group was higher than that in the MSC group (*P* < 0.005). (**B**–**E**) Histopathological analysis of kidneys by haematoxylin–eosin staining and Masson-trichrome staining. Distention of tubular lumens (*P* < 0.01), interstitial fibrosis (*P* < 0.005), and reduction in glomerular numbers (*P* < 0.005) were observed in the control group as compared to those parameters in the MSC group. Data are expressed as the mean ± SEM. Black scale bars: 500 μm, white scale bars: 100 μm.

**Figure 2:**
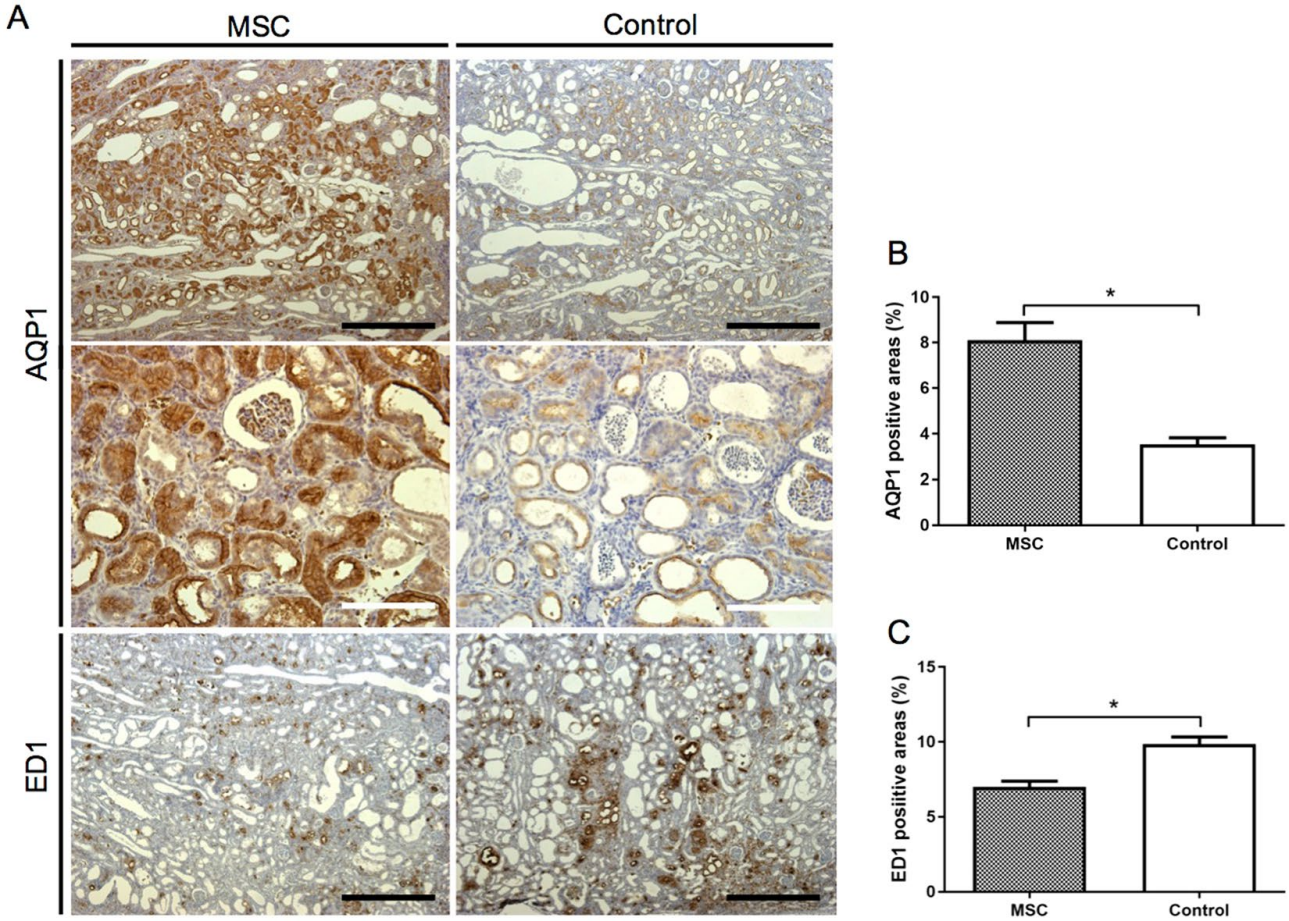
Immunohistopathology of kidneys from adenine-fed rats. (**A**) Immunohistopathological expression of AQP1 and ED1. (**B**) Mesenchymal stem cells (MSCs) transplantation significantly increased the AQP1-positive area (*P* < 0.0001) and (**C**) reduced the ED1-positive area (*P* < 0.0005) as compared to the those in control group. Data are expressed as the mean ± SEM. Black scale bars: 500 μm, white scale bars: 100 μm.

In the first experiment (Experiment 1 in the Materials and Methods), we aimed to assess the effects of MSCs transplantation on the pathology of CKD induced by 4 weeks of an adenine-supplemented diet in SD rats. The baseline characteristics of control and MSCs-administered rats before sacrifice are outlined in Supplementary Table 1. There were no differences between the control and MSC groups. After 4 weeks of the adenine-supplemented diet, the average kidney weight in the control group was higher than that in the MSC group (Fig. 1A). Histopathology of the kidney showed greater dilation of tubular lumens (*P* < 0.01), smaller glomerular numbers (*P* < 0.01), and higher interstitial fibrosis (*P* < 0.005) in the control group than in the MSC group (Fig. 2B–E). Immunohistopathological analysis revealed that MSCs transplantation significantly increased AQP1-positive areas and reduced ED1-positive areas as compared to those with control treatment (Fig. 2).

**Figure 3:**
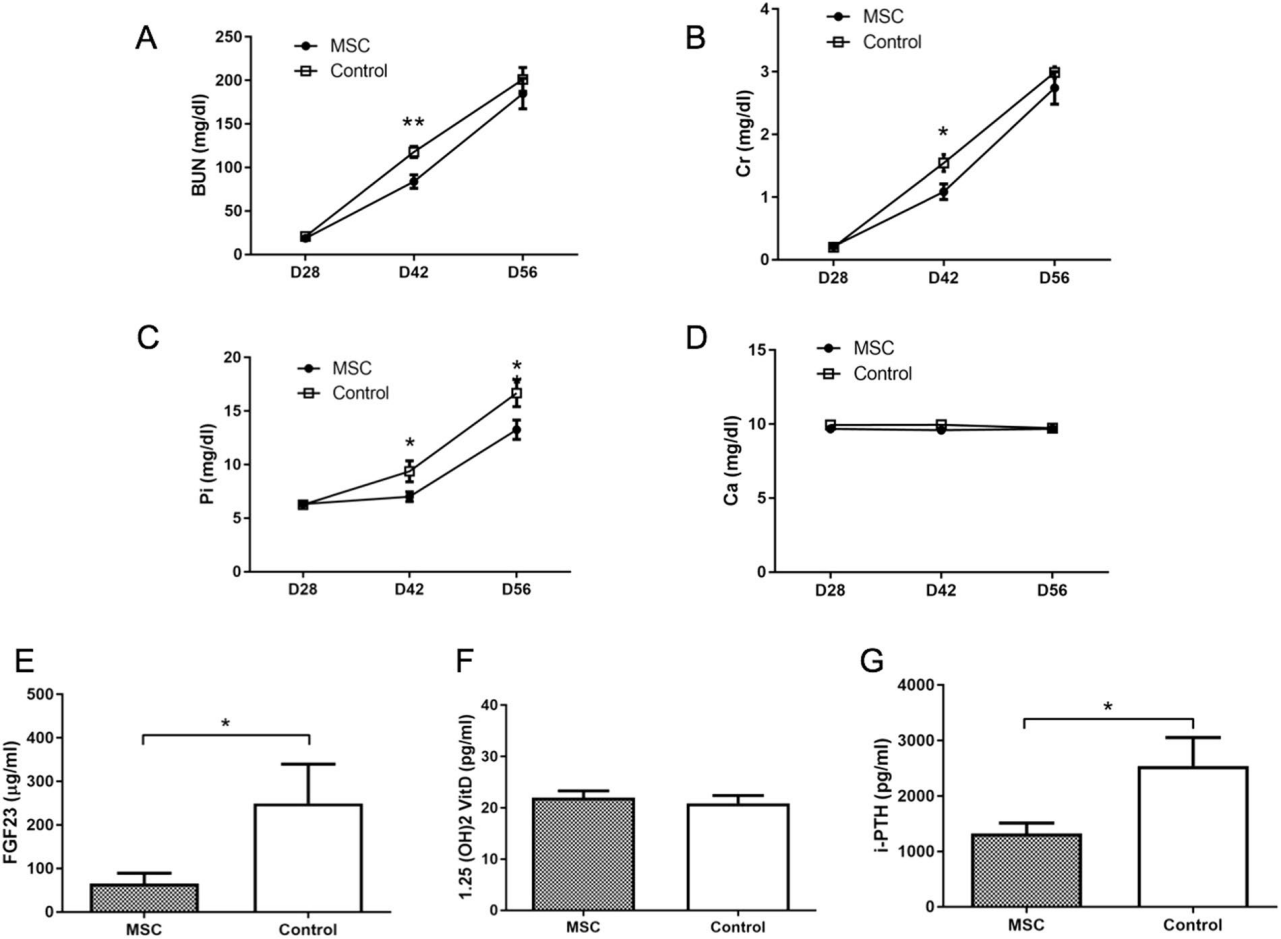
Serum parameters in adenine-fed rats. (**A**–**G**) Serum levels of blood urea nitrogen (BUN), creatinine (Cre), inorganic phosphorus (Pi), and calcium (Ca) in adenine-fed and control rats. (**A**,**B**) Mesenchymal stem cells (MSCs) transplantation significantly reduced serum BUN (*P* < 0.05) and Cr (*P* < 0.05) levels on day 42. (**C**,**D**,**G**) MSCs significantly reduced serum Pi (*P* < 0.05), FGF23 (*P* < 0.05), and intact parathyroid hormone (PTH) (*P* < 0.05) on day 56 as compared to those in the control group. (**D**,**F**). Serum levels of Ca and 1–25(OH)_2_D_3_ on day 56 did not significantly differ between the MSC and control groups. Data are expressed as the mean ± SEM.

**Figure 4:**
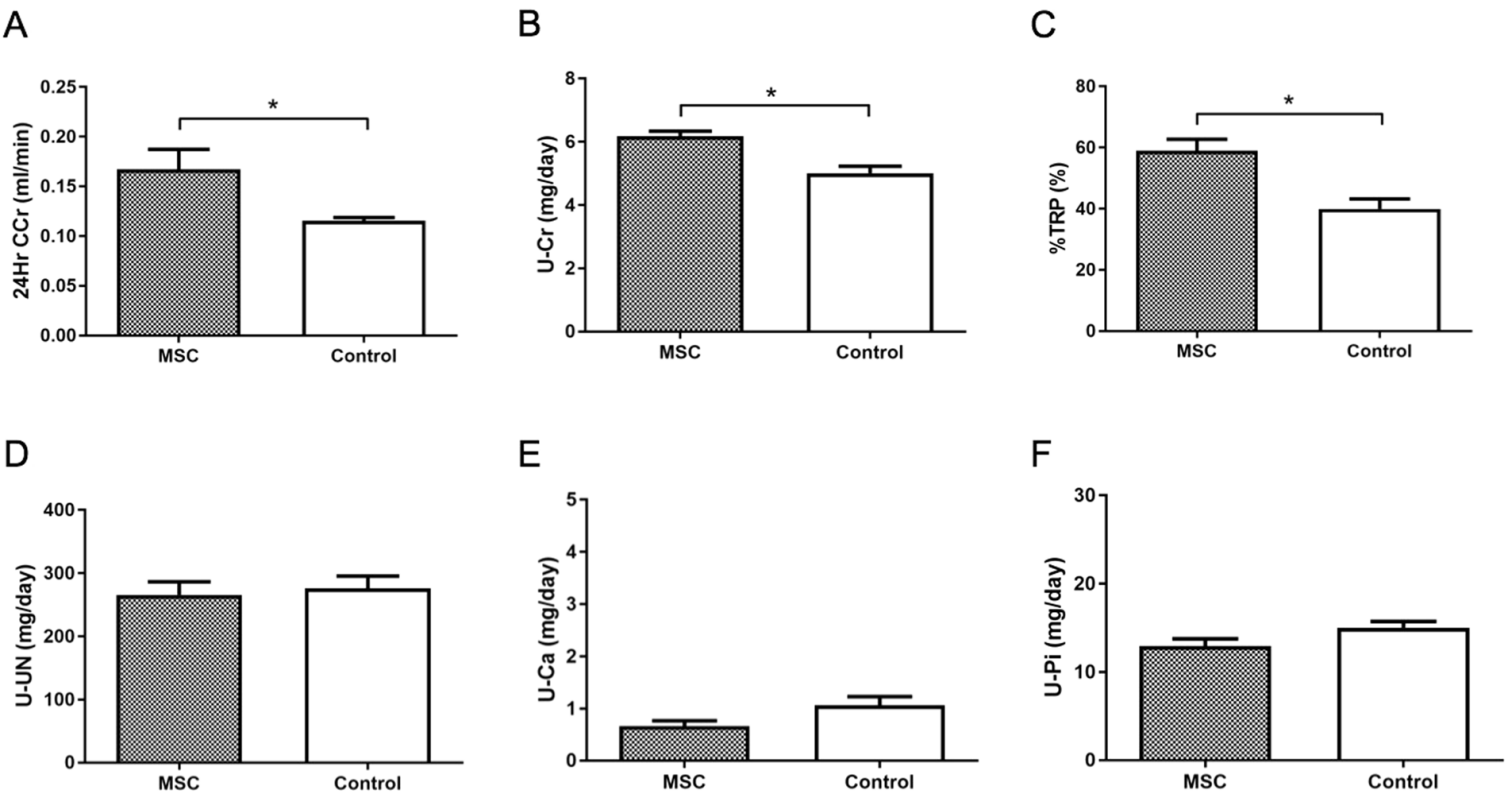
Urinary parameters in adenine-fed rats. (**A**–**C**) Mesenchymal stem cells (MSCs) transplantation significantly elevated 24-h creatinine clearance (24-h CCr) (*P* < 0.05), urine creatinine (Cr) (*P* < 0.005), and the tubular reabsorption of phosphate (%TRP) (*P* < 0.005) on day 56 as compared to those in the control group. (**D**–**F**) Urinary levels of blood urea nitrogen (BUN), calcium (Ca), and inorganic phosphorus (Pi) on day 56 did not significantly differ between the MSC and control groups. Data are expressed as the mean ± SEM.

The levels of blood urea nitrogen (BUN), creatinine (Cr), and inorganic phosphorus (Pi) increased over time with adenine feeding in rats of both groups (Fig. 3A–C). Although the levels of BUN and Cr in the MSC group on day 42 were lower than those in the control group (Fig. 3A, *P* < 0.05), the levels on day 56 did not significantly differ between both groups. However, the level of Pi in the MSC group on day 56 was lower than that in the control group (Fig. 3C, *P* < 0.05). Urinary parameters showed that MSCs transplantation significantly raised 24-hour creatinine clearance (24-h CCr) (*P* < 0.05), urinary Cr excretion (*P* < 0.005), and tubular reabsorption of phosphate (%TRP) (*P* < 0.005) as compared to those with control treatment (Fig. 4A–C). Furthermore, MSCs transplantation significantly decreased serum levels of fibroblast growth factor-23 (FGF23) and intact parathyroid hormone (PTH) levels as compared to those with control treatment (Fig. 3E,G). These results suggested that MSCs transplantation reduces adenine-induced kidney damage including interstitial fibrosis and tubular disorder due to interstitial nephritis, resulting in a reduction in serum Pi.

### MSCs transplantation prevents the progression of vascular calcification

**Figure 5:**
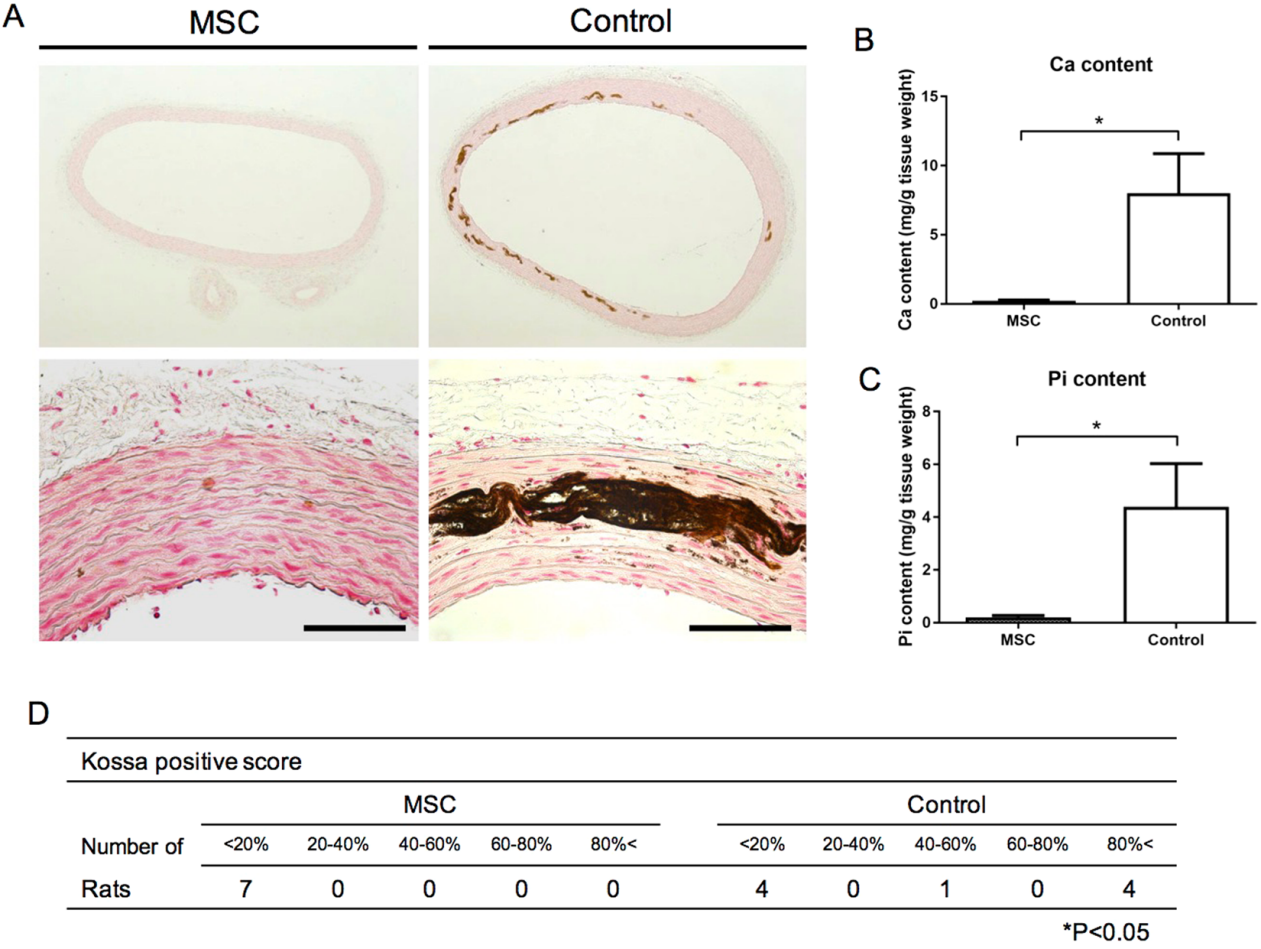
Effect of mesenchymal stem cells (MSCs) transplantation on calcification of the aortic media. (**A**) Von Kossa staining of the thoracic aorta media revealed that MSCs transplantation suppressed vascular calcification as compared to that in the control group. (**B**,**C**) MSCs transplantation reduced calcium (*P* < 0.0005) and phosphorus (*P* < 0.01) contents in the thoracic aorta as compared to those in the control group. (**D**) Calcification scores in the MSC group were significantly lower than those in the control group (*P* < 0.05). Data are expressed as the mean ± SEM. Black scale bars: 100 μm.

**Figure 6:**
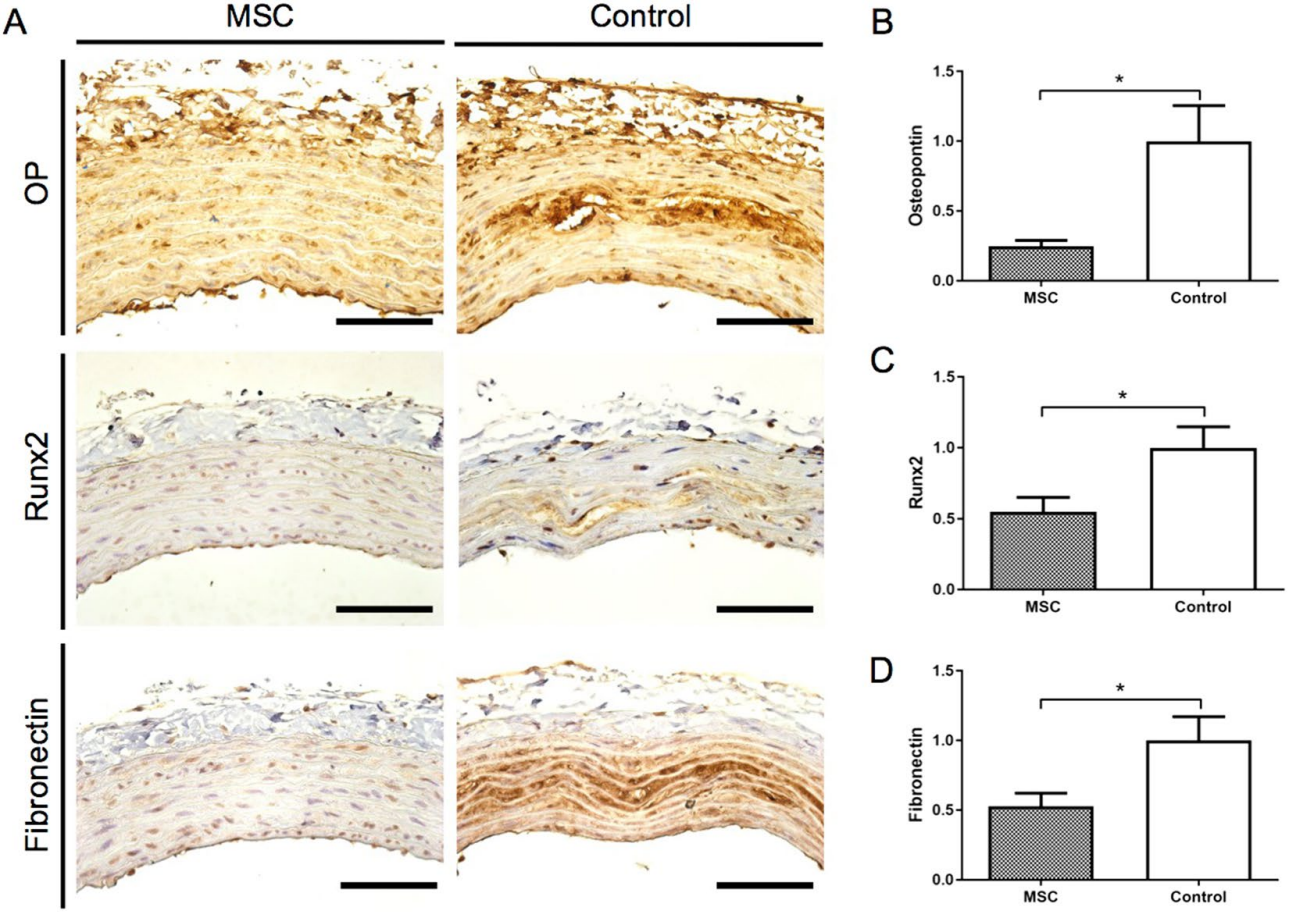
Expression of osteopontin (OP), Runx2, and fibronectin in the thoracic aorta. (**A**) Expression of OP, Runx2, and fibronectin in the thoracic aorta was detected by immunohistochemistry. (**B**–**D**) OP (*P* < 0.05), Runx2 (*P* < 0.05), and fibronectin (*P* < 0.05) mRNA expression were significantly suppressed in the MSC group as compared to the control group. Data are expressed as the mean ± SEM. Black scale bars: 100 μm.

Histopathological von Kossa staining of the thoracic aorta media revealed vascular calcification in the control group, which was suppressed in the MSC group, after 4 weeks of adenine-supplemented diet (Fig. 5A). Calcification scores in the MSC group were significantly lower than those in the control group (Fig. 5D; *P* < 0.05). In the control group, calcium (Ca) and inorganic phosphorus (Pi) levels in the thoracic aorta were elevated; however, the increased Ca and Pi levels seen in the control group were significantly suppressed in the MSC group (Fig. 5B,C; *P* < 0.0005 and *P* < 0.01, respectively). Immunohistochemistry showed reduced expression of osteopontin (OP), Runx2, and fibronectin in the MSC group (Fig. 6A). The mRNA expression of OP (Fig. 6B; *P* < 0.05), Runx2 (Fig. 6C; *P* < 0.05), and fibronectin (Fig. 6D; *P* < 0.05) was also significantly suppressed in the MSC group as compared to that in control rats.

### Injected MSCs are not detected in the damaged kidney and aorta

**Figure 7:**
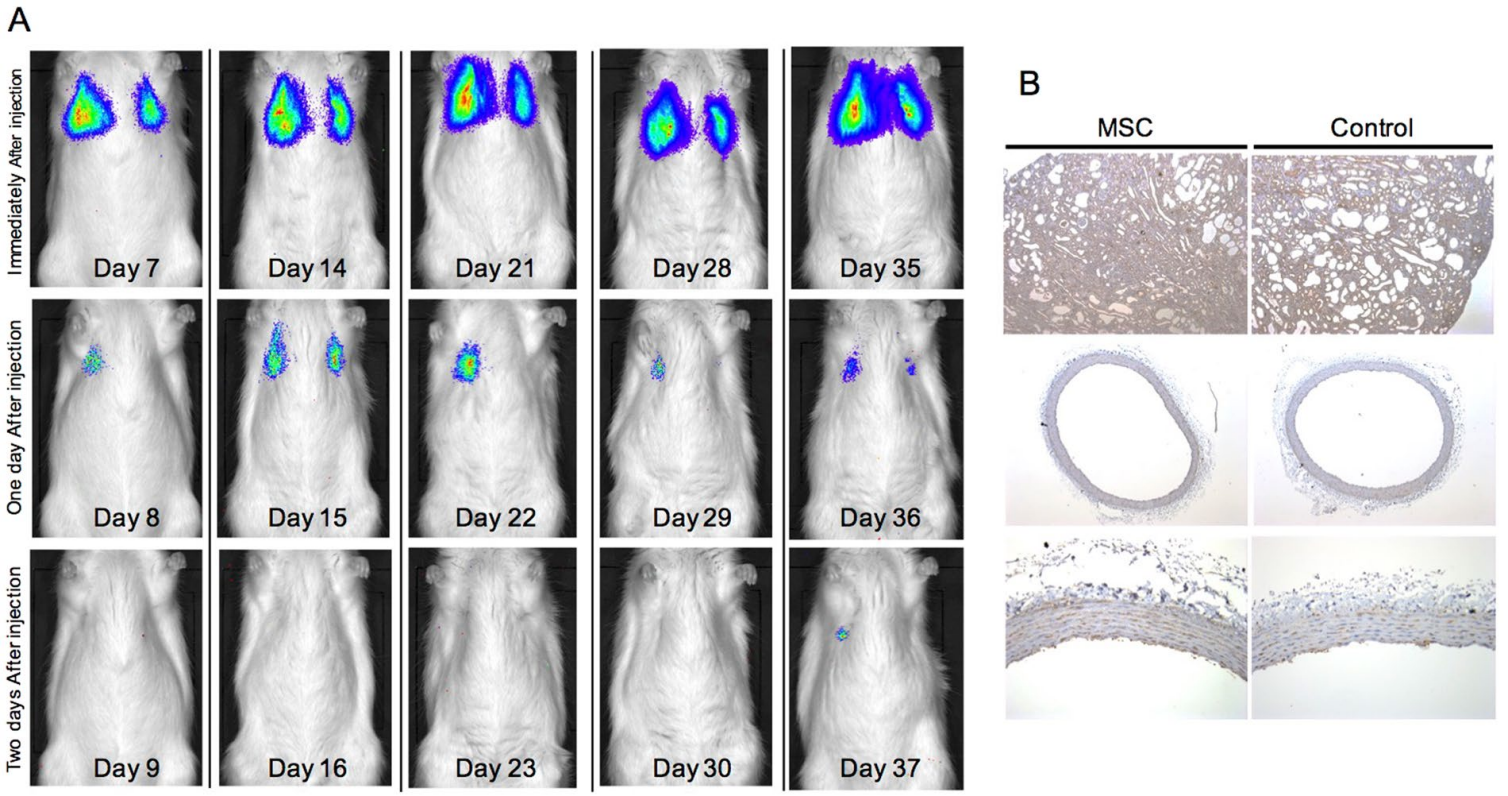
*In vivo* imaging of luciferase positive (Luc+) mesenchymal stem cells. (**A**) Luminescence was observed bilaterally in the lungs, but not in the kidneys. The signal disappeared on the third day. (**B**) Expression of luciferase was not detected by immunohistochemistry.

In Experiment 2, we used luciferase-expressing (Luc+) MSCs to assess the distribution of MSCs *in vivo*. Luc+ MSCs accumulated only in the lungs, likely as a result of being trapped in the pulmonary capillaries (Fig. 7A). The Luc signal disappeared within two days after transplantation. Luc+ MSCs did not home to damaged kidneys or the thoracic aorta via the lungs. Immunohistochemical staining confirmed that Luc+ MSCs did not migrate into the injured kidneys and thoracic aorta at the time of sacrifice (Fig. 7B). These results suggested that the therapeutic effect of MSCs on adenine-induced kidney damage is not directly because of the differentiation of MSCs, but rather is mediated by the metabolic function of MSCs.

### Ca and Pi levels in the thoracic aorta significantly correlate with serum Ca and Pi levels

**Figure 8:**
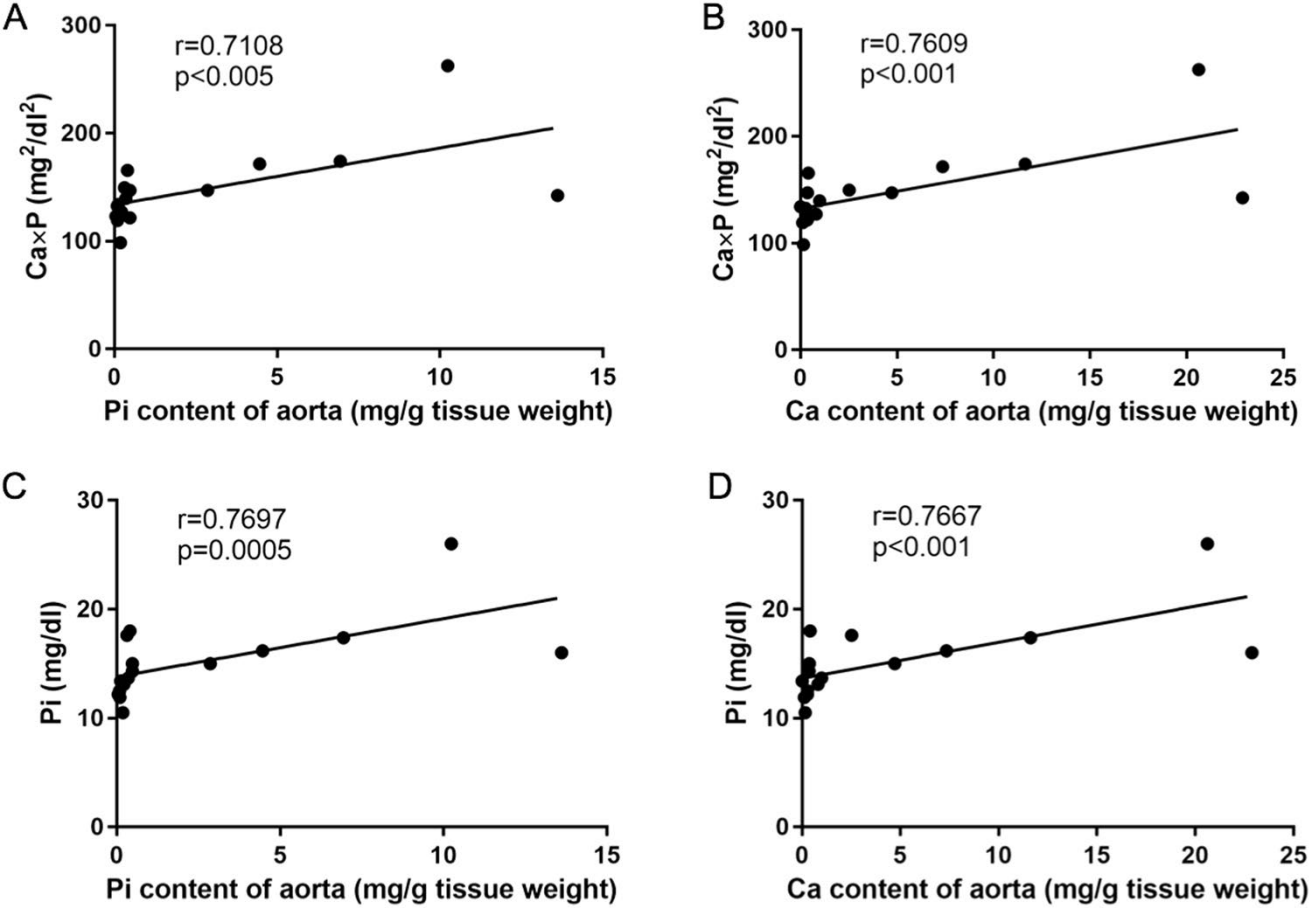
Correlation between the inorganic phosphorous (Pi) or calcium (Ca) content of the thoracic aorta and serum Pi or the Ca × Pi product at time of sacrifice. (**A**,**B**) Linear correlation between the Pi or Ca content of the thoracic aorta and the Ca × Pi product at the time of sacrifice (*P* < 0.005, *P* < 0.001, respectively). (**C**,**D**) Linear correlation between the Pi or Ca content of the thoracic aorta and serum Pi (*P* < 0.0005, *P* < 0.001, respectively).

We next analyzed the correlation between Ca and Pi levels in the thoracic aorta and serum Ca and Pi levels at the time of sacrifice in Experiment 1. Pi and Ca levels in the aorta significantly correlated with the Ca × Pi product and serum Pi levels at the time of sacrifice (Fig. 8). This suggested that the inhibitory effect of MSCs transplantation on vascular calcification depends on serum Pi levels.

## Discussion

In this study, we analyzed the effects of autologous MSCs transplantation on the progression of kidney damage and vascular calcification in an adenine-induced CKD model. We found that MSCs transplantation reduced adenine-induced kidney injury and the progression of vascular calcification, suggesting that this could represent a promising treatment option for patients with CKD.

Cardiovascular disease (CVD) is a major cause of morbidity and mortality for CKD patients waiting for kidney transplantation, and it is the most common cause of death in transplant recipients^29^. Many nephrologists pay strive to prevent CVD in CKD patients to improve long-term outcomes^30^. Therefore, new modalities to prevent the progression of vascular calcification are urgently needed. In this study, we demonstrated for the first time that MSCs transplantation reduces vascular calcification using an adenine-induced CKD model. If this therapy were to be clinically applied, it might contribute to improved prognosis for not only CKD patients, but also kidney transplant recipients.

Vascular calcification in CKD is thought to be due to the induction of osteoblastic phenotypic changes, which result from several factors including not only hyperphosphatemia, but also uremic toxins, oxidative stress, and haemodynamic state^31^. Inhibition of these factors has been shown to effectively prevent vascular calcification^32–36^. In adenine-induced CKD rats, vascular calcification generally correlates with the serum Ca × Pi product^26,37^ and can be decreased by administering phosphorus-binding agents^32–34^. In our study, Pi and Ca levels in the aorta significantly correlated with the Ca × Pi product, as well as with serum Pi levels, as previously reported^26,37^. Serum Pi levels in the MSC group were lower than those in the control group. Therefore, these results suggest that the suppression of kidney damage and improvement of kidney function lead to a decline in the serum Pi levels, and that the Ca × Pi product depends on residual kidney function, resulting in amelioration of vascular calcification.

A recent study showed that Gli1+ MSC-like cells are critical adventitial progenitors for vascular remodelling^22^. Thus, we analyzed whether injected MSCs migrate into the media using Luc+ MSCs. The results showed that injected MSCs did not migrate into the media, suggesting that MSCs transplantation does not negatively affect vascular calcification during CKD.

MSCs additionally inhibit oxidative stress^14,29^, which is reportedly associated with vascular calcification^38,39^. We did not examine the effect of MSCs transplantation on the oxidative stress of vessels or the hemodynamic state. Moreover, there is a possibility that soluble factors or microvesicles derived from MSCs directly attenuate pre-existing vascular calcification. However, we did not generate data regarding the effect of MSCs on pre-existing vascular calcification. Therefore, further studies are needed to determine the exact mechanism through which MSCs transplantation affects vascular calcification.

Studies using various renal injury models have demonstrated the ability of MSCs to repair kidney damage and attenuate renal fibrosis through modulation of the immune system^40^, the reduction in oxidative stress^14,41^, the secretion of soluble factors such as hepatocyte growth factor (HGF) and vascular endothelial growth factor (VEGF)^17–19,21^, and microvesicles^20,41^. Our study showed that injected Luc+ MSCs do not migrate into the damaged kidneys, suggesting that the mechanism for kidney repair in this study involves soluble factors (e.g., VEGF) as previously reported^21^ or microvesicles derived from the MSCs. In contrast, the serum levels of Cr at the time of sacrifice were not significantly different between MSCs-treated and control rats in our study. A recent study showed that adenine induces interstitial nephritis and polyuria, resulting in dehydration^2^; thus, to evaluate the degree of dehydration, we analyzed hematocrit (Ht) levels in rats on day 42. These were higher in the MSCs-treated group than in the control group (Supplementary Fig. 2, *P* < 0.005), suggesting that the former group was more affected by dehydration. In contrast, urinary parameters indicated that 24-h CCr and urinary Cr excretion were significantly higher in the MSCs-treated group than in the control group (Fig. 4A,B). From these results, we suggest that dehydration induced an elevation in serum Cr levels in the MSCs-treated group, resulting in the discrepancy between pathology and serum Cr levels in this study.

In conclusion, to our knowledge, we demonstrated for the first time that MSCs transplantation has beneficial effects on vascular calcification in adenine-induced CKD rats. The renoprotective effect of MSCs is regarded as one of the mechanisms through which vascular calcification is suppressed. Our findings suggest that MSCs transplantation has potential as a new therapeutic strategy for the treatment of vascular calcification in patients with CKD.

## Materials and Methods

### Ethics statement

All experimental procedures were approved by the Animal Committee of the Jikei University School of Medicine and conducted according to the Fundamental Guidelines for Proper Conduct of Animal Experiments and Related Activities in Academic Research Institutions issued by the Japanese Ministry of Education, Culture, Sports, Science and Technology. All surgeries were performed under isoflurane anaesthesia and all efforts were made to minimize suffering.

### Experimental protocols

#### Experiment 1

Adult male SD rats (9 weeks of age) were purchased from Japan SLC (Hamamatsu, Japan) and were fed a standard CE-2 diet (Nihon CLEA, Tokyo, Japan). They were kept in cages in pairs and allowed free access to food and water. Adipose tissue was dissected from around the groin area and the femur of rats (day 1). After 4 weeks (at 13 weeks of age), the rats were divided into two groups: an adenine control group (n = 9) and an MSC group (n = 7), and were fed a CE-2 diet containing 0.75% adenine (Wako, Osaka, Japan) for 4 weeks. Following adenine feeding, rats were injected with 5 × 10^5^ MSCs resuspended in 1 ml of PBS or vehicle control (1 ml of PBS) via the tail vein every week (days 28, 35, 42, 49, and 56). Blood was sampled from the tail vein every two weeks (days 28, 42, and 56). The rats were placed in metabolic cages for 24-h urine collection on day 56. After 4 weeks of the adenine diet (at 17 weeks of age), adenine-treated rats were returned to a normal diet. After 2 days of normal diet (day 58), all animals were sacrificed by abdominal aortic puncture under isoflurane anaesthesia. The experimental design is summarized in Supplementary Fig. S3.

#### Experiment 2

Adult male Lewis rats (10 weeks old) were purchased from Japan SLC (Hamamatsu, Japan) and were fed a standard CE-2 diet (Nihon CLEA). After 1 week (at 11 weeks of age), the rats were divided into two groups: an adenine control group (n = 4) and a Luc-MSC group (n = 4), and were fed a CE-2 diet containing 0.75% adenine (Wako, Osaka, Japan) for 4 weeks. Following adenine feeding, the rats were injected with 5 × 10^5^ luciferase-expressing (Luc+) MSCs resuspended in 1 ml of PBS or vehicle control (1 ml of PBS) via the tail vein every week (days 7, 14, 21, 28, and 35). After 4 weeks of the adenine diet (at 15 weeks of age), adenine-treated rats were returned to a normal diet. After 1 week of the normal diet (day 42), all animals were sacrificed by abdominal aortic puncture under isoflurane anesthesia. The experimental design is summarized in Supplementary Fig. S4.

### MSCs culture

MSCs were isolated as previously described^27,42^. Briefly, adipose tissues were minced using scissors and washed with phosphate-buffered saline (PBS) (Thermo Fisher Scientific, Waltham, MA, USA) and were then enzymatically dissociated with 1 ml of 0.1% collagenase (type I) (Wako, Osaka, Japan) in PBS for 1 h at 37 °C. The dissociated tissue was incubated in α-minimum essential medium (αMEM) (Thermo Fisher Scientific) supplemented with foetal bovine serum (FBS) (Thermo Fisher Scientific), and centrifuged. The cell pellet was resuspended in PBS. After centrifugation, adipose cells including MSCs were obtained. Luc+ MSCs were obtained from adipose tissues of luciferase transgenic rats^6,43^. Isolated cells were cultured in αMEM supplemented with 20% embryonic stem cell-qualified FBS (Thermo Fisher Scientific).

### Induction of adipogenesis, osteogenesis, and chondrogenesis

Multilineage differentiation of MSCs under adipogenic, chondrogenic, and osteogenic differentiation conditions was analyzed as previously described^27^. Briefly, MSCs were seeded in induction medium with MSC differentiation kits for adipogenic, osteogenic, or chondrogenic induction (BulletKit; Lonza, Walkersville, MD). MSCs were maintained in culture according to the manufacturer’s protocols.

### Flow cytometry

Cells were harvested from culture by treatment with 0.05% trypsin-EDTA (Sigma-Aldrich, St Louis, MO) for 3 min at 37 °C, and approximately 1 × 10^6^ cells were added to tubes and centrifuged at 400 × *g* for 5 min. The pellet was washed twice in ice-cold PBS containing 2% FBS and cells were resuspended at 1 × 10^5^ cells/antibody test. The expression of specific MSCs markers was evaluated by flow cytometry (MACSQuant; Miltenyi Biotec, Gladbach, Germany) using antibodies against CD29 (BD, New Jersey, USA), CD31 (Thermo Fisher Scientific, Waltham, MA, USA), CD45 (R&D systems, MN, USA), and CD90 (BD Biosciences Pharmingen, Tokyo, Japan).

### Bioluminescence imaging

Luc+ MSCs (5 × 10^5^ per rat) from luciferase-transgenic rats were injected into the Luc-MSC group rats via the tail vein every week. Rats were anaesthetized with 2.0% (vol/vol) isoflurane, injected intravenously with 1 ml d-luciferin (33 mg/ml in PBS; Summit Pharmaceuticals International Corp., Tokyo, Japan) and imaged for 10 min on days 0 (1 h post Luc+ MSCs injection), day 1 (24 h post Luc+ MSCs injection), and day 2 (48 h post Luc+ MSCs injection) using the Xenogen IVIS 200 system (Xenogen, Alameda, CA). The experimental design is summarized in Supplementary Fig. S4.

### Blood and urinary biochemistry

Blood and urine samples were analyzed as previously described^26^. Briefly, serum Pi, Ca, BUN, and Cr levels were measured using an automated analyzer (FDC-3500; Fuji Film, Tokyo, Japan). Urinary Cr, Pi, and Ca levels were analyzed according to the manufacturer’s instructions (SRL, Tokyo, Japan). Serum concentrations of PTH and FGF23 were determined by enzyme-linked immunosorbent assay (ELISA) with a rat intact PTH ELISA kit (Immutopics, Inc., San Clemente, CA) and FGF-23 ELISA kit (Kainos Laboratories Inc., Tokyo, Japan). The serum levels of 1-α25-dihydroxyvitamin D3 (1–25(OH)_2_D_3_) were measured using a radioimmunoassay according to the manufacturer’s instructions (SRL).

### Histopathological examinations

For histopathological examination, all resected tissues were fixed with 10% buffered formalin, and 5-μm-thick sections were stained with haematoxylin–eosin. Adipocytes differentiated from MSCs were stained with Sudan III. Osteoblasts differentiated from MSCs were stained by the von Kossa method. Chondrocytes differentiated from MSCs were stained with Safranin O, Fast green, and Toluidine blue using a Cartilage Staining Kit (Takara Bio, Tokyo, Japan)^27,42^. Kidney sections were stained using the Masson trichrome method. The dimensions of tubular lumen dilatation and the degree of interstitial fibrosis, as well as the glomerular numbers were quantified from 10 high-power fields of the cortical area per section using MetaValue software (Molecular Devices, Sunnyvale, CA) and a BIOREVO BZ-9000 microscope (Keyence, Osaka, Japan) as previously described^7^. The kidney sections were also examined immunohistochemically for the expression of ED-1 (ab31630; Abcam, Cambridge, UK), AQP 1 (sc-20810; Santa Cruz Biotechnology, Inc, Dallas, TX), and luciferase (PM016; MBL, Nagoya, Japan). Areas of positive staining were quantified using MetaValue. Aorta sections were stained using the von Kossa method and scored semi-quantitatively for Kossa-positive areas using a previously described scoring system^26^ with a slight modification. Aortic tissue was also examined for the expression of osteopontin (LB4225; LSL, Tokyo, Japan), Runx2 (sc-8566; Santa Cruz Biotechnology, Inc), fibronectin (sc-9068; Santa Cruz Biotechnology, Inc), and luciferase (MBL) using monoclonal antibodies.

### Ca and Pi contents in thoracic aorta

The levels of Ca and Pi in the aorta were determined as described previously^26^. Briefly, thoracic aortas were weighed shortly after removal and were subsequently extracted with 150 mM HCl overnight at room temperature to dissolve any minerals. The levels of Ca and Pi in the solution were analysed according to the manufacturer’s instructions (Wako, Osaka, Japan). The levels of Ca and Pi in the aorta were calculated as the weight of calcium or phosphorus per wet tissue weight of aorta.

### RNA isolation and real-time reverse transcriptase polymerase chain reaction (qRT-PCR)

RNA was extracted from tissues using TRIzol Reagent (Thermo Fisher Scientific) according to the manufacturer’s instructions. Genomic DNA was removed using DNase I (Takara Bio, Otsu, Japan) and cDNA was synthesized from the total RNA using the Superscript RT-PCR system. Osteopontin, Runx2, and fibronectin mRNA levels were semi-quantified by qRT-PCR using an ABI7000 (Thermo Fisher Scientific) with RT2 SYBR Green Master Mix (Qiagen, Hilden, Germany) and a Rat osteogenesis PCR Array (Qiagen) according to the manufacturer’s instructions.

### Statistical analysis

Data are presented as the mean ± SEM. Statistical analyses were carried out using GraphPad Prism 5 (GraphPad Software, San Diego, CA). The significance of differences between two mean values was determined using unpaired *t*-tests. The Pearson correlation coefficient was used to evaluate the correlation between the Ca and P contents of the aorta and serum Ca × P products. Statistical significance was defined as *P* < 0.05.

## Electronic supplementary material

Supplementary Information
