# Supplementary material for: Effect of adipose-derived mesenchymal stem cell transplantation on vascular calcification in rats with adenine-induced kidney disease
Source: Sci Rep. 2017 Oct 25;7:14036. doi: 10.1038/s41598-017-14492-9 (PMC5656613; doi:10.1038/s41598-017-14492-9)
Supplement: Supplementary file 1 — Supplementary Information [file 41598_2017_14492_MOESM1_ESM.pdf]

Supplementary Information

Effect of adipose-derived mesenchymal stem cell transplantation on vascular calcification in rats with adenine-induced kidney disease

Shinya Yokote [a,*], Yuichi Katsuoka [b], Akifumi Yamada [c], Ichiro Ohkido [a], Takashi Yokoo [a]

[a] Division of Nephrology and Hypertension, Department of Internal Medicine, The Jikei University School of Medicine, 3-25-8 Nishi-shinbashi, Minato-ku, Tokyo 105-8461, Japan

[b] Department of Urology, St. Marianna University School of Medicine, 2-16-1, Sugao, Miyamae- ku, Kawasaki, Kanagawa, 216-8511, Japan

[c] Department of Pediatrics, The Jikei University School of Medicine, Tokyo, Japan

*Correspondence and requests for materials should be addressed to T.Y. (e-mail: tyokoo@jikei.ac.jp)

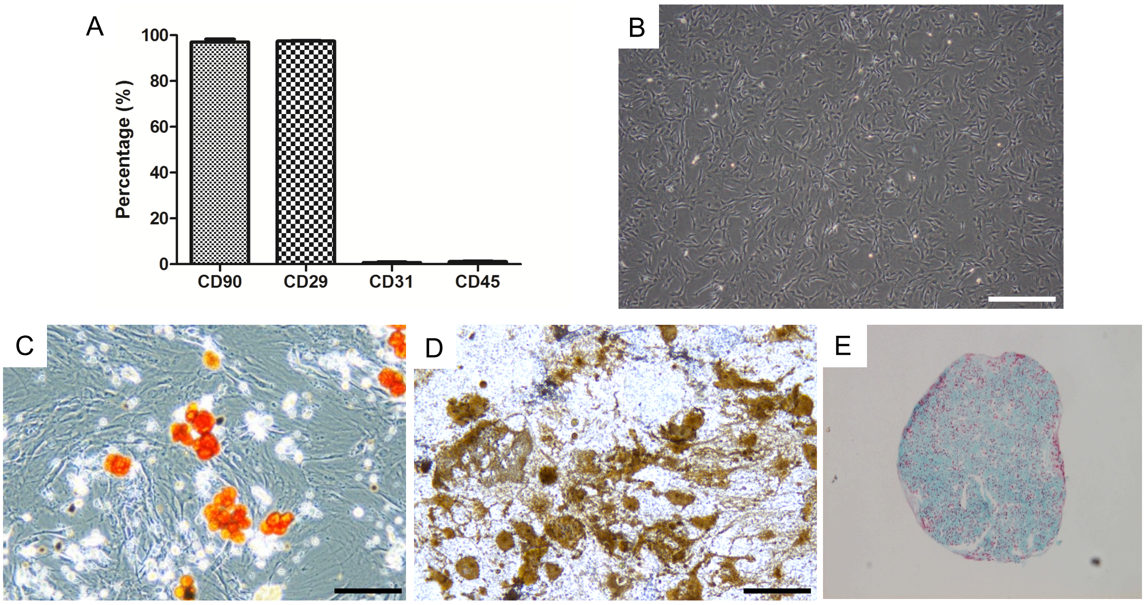

Figure S1. Cell surface markers and differentiation capacities of mesenchymal stem cells (MSCs)

(A) MSCs were positive for CD90 and CD29, and negative for CD31 and CD45. Data are the mean ± S.E.M. (B–E) MSCs had the capacity to differentiate into adipogenic, chondrogenic, and osteogenic lineages. (Scale bars: 500 μm in B; 100 μm in C and D).

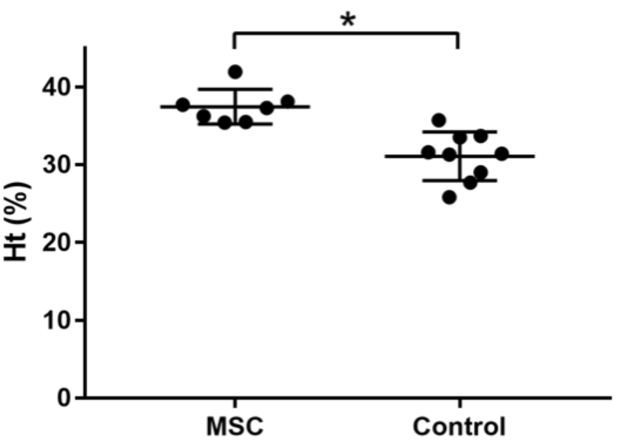

Figure S2. Hematocrit (Ht) in adenine-fed rats on Day 42.

Average of Ht levels on Day 42 in the MSC group were higher than those in the control group ($P < 0.005$). Data are expressed as the mean ± SEM.

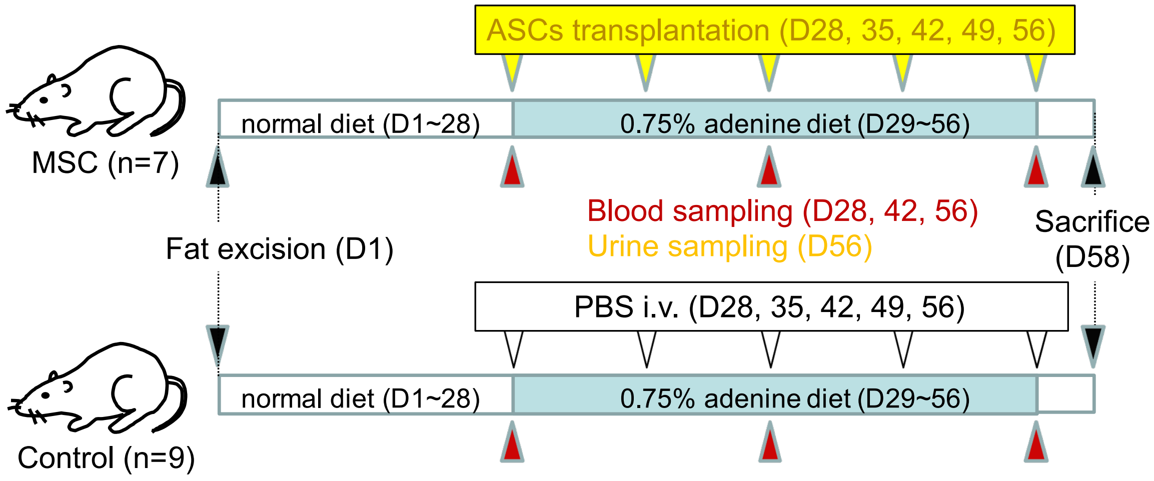

Figure S3. Experimental set-up of Experiment 1.

Adipose-derived mesenchymal stem cells (ASCs) were obtained from fat around the left femurs of 9-week-old male SD rats. Chronic kidney disease (CKD) was induced by maintaining the rats on a 0.75% adenine-supplemented diet for 4 weeks. The rats were randomized into two groups: an adenine control group (n = 9) and an MSC group (n = 7). ASCs (5×10^5/rat) were intravenously administrated to MSC rats every week for 4 weeks. All rats were sacrificed on day 58.

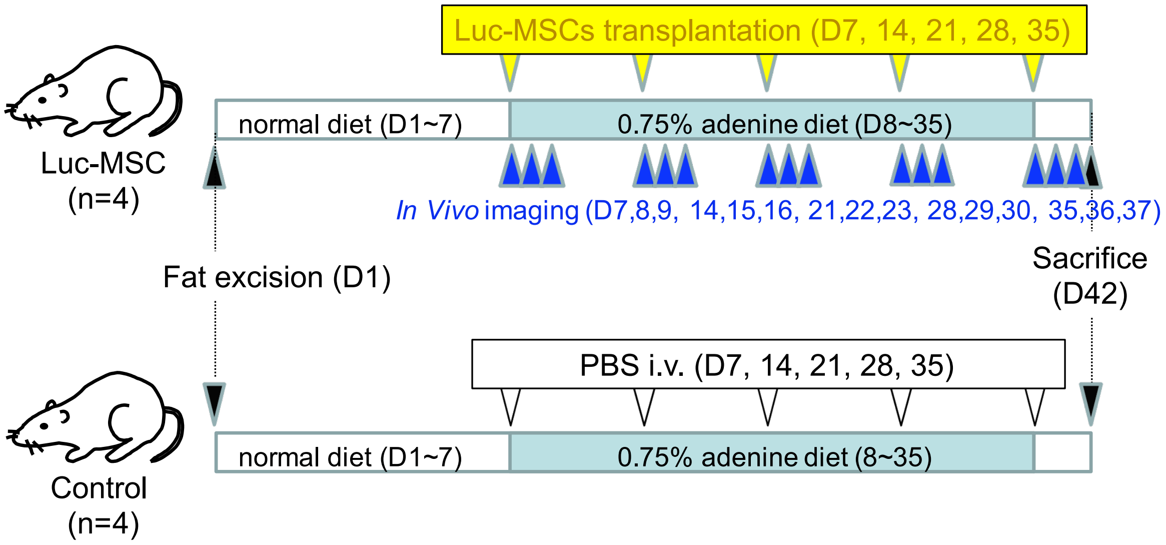

Figure S4. Experimental set-up of Experiment 2.

Luc+ mesenchymal stem cells (MSCs) were obtained from luciferase-transgenic rats. Chronic kidney disease (CKD) was induced by maintaining the rats on a 0.75% adenine-supplemented diet for 4 weeks. The rats were randomized into two groups: an adenine control group (n = 4) and a Luc+ MSC group (n = 4). Luc+ MSCs (5×10^5/rat) were intravenously administrated to Luc-MSC rats every week for 4 weeks. Rats were imaged 10 min after D-luciferin injection using the Xenogen IVIS 200 system on days 0 (1 h post Luc+ MSCs injection), 1 (24 h post Luc+ MSCs injection), and 2 (48 h post Luc+ MSCs injection).

Table S1. Physical parameters at time of sacrifice for adenine-fed versus control rats

	Body weight (g/day)	Water intake (g/day)	Urine volume (g/day)	Food consumption (g/day)	Stool (g/day)
MSC (n=7)	282±3.48	61.5±2.86	46.6±3.21	11.1±0.68	3.3±0.32
Control (n=9)	272±7.63	52.8±4.41	45.8±3.28	9.96±1.36	3.8±0.48
P value	n.s.	n.s.	n.s.	n.s.	n.s.

n.s., not significant.
